# Irisin, Energy Homeostasis and Male Reproduction

**DOI:** 10.3389/fphys.2021.746049

**Published:** 2021-09-21

**Authors:** Pallav Sengupta, Sulagna Dutta, Ivan Rolland Karkada, Roland Eghoghosoa Akhigbe, Suresh V. Chinni

**Affiliations:** ^1^Physiology Unit, Faculty of Medicine, Bioscience and Nursing, MAHSA University, Kuala Lumpur, Malaysia; ^2^Department of Oral Biology and Biomedical Sciences, Faculty of Dentistry, MAHSA University, Kuala Lumpur, Malaysia; ^3^Department of Physiology, Ladoke Akintola University of Technology, Ogbomoso, Nigeria; ^4^Reproductive Biology and Toxicology Research Laboratories, Oasis of Grace Hospital, Osogbo, Nigeria; ^5^Department of Biotechnology, Faculty of Applied Sciences, AIMST University, Bedong, Malaysia

**Keywords:** energy metabolism, irisin, male infertility, reproduction, metabolic syndrome

## Abstract

Irisin is a novel skeletal muscle- and adipose tissue-secreted peptide. It is conventionally regarded as an adipomyokine and is a cleaved fragment of Fibronectin type III domain-containing protein 5 (FNDC5). It is involved in the browning of white adipose tissue, glucose tolerance, and reversing of metabolic disruptions. Fertility is closely linked to energy metabolism and the endocrine function of the adipose tissue. Moreover, there is established association between obesity and male infertility. Irisin bears strong therapeutic promise in obesity and its associated disorders, as well as shown to improve male reproductive functions. Thus, irisin is a molecule of great interest in exploring the amelioration of metabolic syndrome or obesity-induced male infertility. In this review we aim to enumerate the most significant aspects of irisin actions and discuss its involvement in energy homeostasis and male reproduction. Though current and future research on irisin is very promiscuous, a number of clarifications are still needed to reveal its full potential as a significant medicinal target in several human diseases including male infertility.

## Introduction

Metabolic syndrome and energy dyshomeostasis are among the major disruptors of male reproductive health (Morrison and Brannigan, 2015). Obesity links with male infertility by multitudinous mechanisms (Katib, 2015; Bhattacharya et al., 2020). Men with metabolic disorders often have disturbed levels of reproductive hormones, most prominently with low testosterone and high estradiol (Pauli et al., 2008). These hormonal imbalance reportedly corresponds to the severity of metabolic syndrome (Rosenblatt et al., 2015). Insulin Resistance (IR), inflammation and Oxidative Stress (OS) also are underlying players in the mechanism how excess body fat disrupts reproductive functions (Morrison and Brannigan, 2015).

Various classical and non-classical hormones and factors have been discussed in bridging the knowledge gap among metabolic disorders, energy dyshomeostasis and male infertility (Alahmar et al., 2019; Bhattacharya et al., 2019; Dutta et al., 2019a,b, c; İrez et al., 2019; Sengupta et al., 2019a,b). Irisin, discovered by Boström et al. (2012), is a novel myokine/adipokine secreted by skeletal muscle as well as adipose tissues. Irisin is a remarkable molecule which is mainly induced via exercise, and in the adipose tissues, it converts white adipocytes into metabolically active brown adipocytes, thereby holding promise as a therapeutic in obesity (Zhang et al., 2014, 2016b). Irisin has been shown to improve insulin sensitivity, enhancing cognitive capacities, thereby reversing metabolic imbalances and associated disorders (Perakakis et al., 2017). Despite its essential contribution in energy homeostasis, its detailed physiological actions on reproduction are yet to be explored. Few studies on animals indicate positive impact of irisin upon male fertility (Nanees and Reham, 2018; Tekin et al., 2019; Luo et al., 2021). Thus, it may be assumed that irisin, via reversing obesity, also ameliorates the obesity-induced disruptions in male fertility. The present article thus reviews the available literature pertaining to the versatile roles of irisin in metabolism, energy homeostasis and male reproduction and discusses the possible involved mechanisms.

## Irisin: An Adipo-Myokine and Its Receptors

The precursor of irisin is a transmembrane glycoprotein, Fibronectin type III domain-containing protein 5 (FNDC5) which was detected for the first time in 2002 (Ferrer-Martínez et al., 2002; Teufel et al., 2002), and is also called FRCP2 and Pep. The FNDC5 proteolytic cleavage produces irisin in response to Peroxisome proliferator-activated receptor gamma coactivator 1 alpha (PGC-1 α) activation (Boström et al., 2012). Irisin bears a molecular weight of 12 kDa with 112 amino acid residues (Boström et al., 2012), while the structure of irisin is yet to fully revealed.

Irisin was discovered through a study in search of factors secreted in response to PGC-1α by the skeletal muscles (Schumacher et al., 2013). PGC-1α mediates the physiological benefits of exercise such as white-to-brown fat conversion (Handschin and Spiegelman, 2008), improvement of insulin sensitivity and signaling (Wenz et al., 2009). Studies have also demonstrated that irisin is primarily released in response to exercise (Boström et al., 2012; Huh et al., 2012).

Barely 6 years after the discovery of irisin, Kim et al. (2018) clearly demonstrated that the physiological actions of irisin are mediated via αV integrins located in osteocytes, myocytes, and adipose tissues. They showed that irisin therapy ameliorated hydrogen peroxide-induced apoptosis in MLO-Y4 (osteocyte-like) cell-line, demonstrating that irisin confers protection against apoptosis and induces bone resorption by upregulating sclerostin (Kim et al., 2018). In addition, they demonstrated that FNDC5-knockout mice had significantly lower expression of receptor activator of nuclear factor kappa-B ligand (RANKL) mRNA (Kim et al., 2018). Quantitative proteomics analyses in MLO-Y4 osteocytes identified five cell surface proteins as possible receptor candidates for irisin. Among them, only integrin β1 which binds with α-integrins to form obligate heterodimers, is known to trigger downstream signaling; phosphorylation of focal adhesion kinase (FAK), protein kinase B (AKT), and cyclic AMP (cAMP) response element-binding protein (CREB) (Schaller et al., 1994; Giancotti and Ruoslahti, 1999; D’Amico et al., 2000). Irisin-treated MLO-Y4 cells showed phosphorylation of FAK, AKT, CREB, and Zyxin (Kim et al., 2018). This infers that irisin activates a pathway of integrin-like signaling. The αV/β5 integrin had the highest binding affinity while other integrin complexes showed weak binding to irisin. Their quantitative proteomics with mass spectrometry revealed that αV is the most abundant integrin protein in MLO-Y4 cells followed by integrin β1, integrin α5, integrin β5, integrin β3, integrin β6, and integrin β8. Furthermore, it was reported that HEK293T cells with forced expression of integrin αV/β5 but not αV/β3 showed enhanced FAK phosphorylation with irisin treatment. Inhibition of αV/β5 absolutely blocked the observed irisin-driven phosphorylation of FAK, CREB, and Zyxin. This finding highlights the role of integrin αV/β5 in irisin-mediated functions (Kim et al., 2018).

**FIGURE 1:**
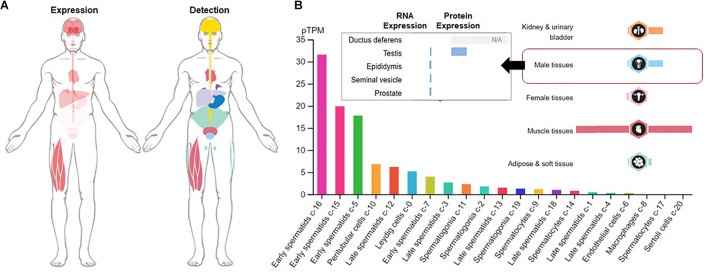
Expression and detection of Fibronectin type III domain containing 5 (FNDC5). **(A)** Global expression and detection of FNDC5 RNA/protein; **(B)** FNDC5 RNA (normalized expression) and protein expression in overall male reproductive tissues and specific testicular cells.

Although the physiological roles of irisin are still evolving, recent studies have shown that irisin secretion is not limited to the osteocyte, myocytes and adipose tissue as it has been found in a variety of tissues. In central nervous system (CNS), irisin expression has been detected in the Purkinje cells of the cerebellum (Dun et al., 2013), spinal cord and cerebral cortex (Huh et al., 2012). In peripheral tissues, it is reported in liver, kidney (Aydin, 2014), salivary glands (Aydin et al., 2013), cardiac muscles (Aydin et al., 2017), skin and testis (Aydin et al., 2014). Among the male reproductive tissues, testis bears the highest irisin expressions followed by prostate gland, while within testis, irisin is mostly expressed in the developing germ cells, peritubular cells and Leydig cells (The Human Protein Atlas, 2021; Figure 1).

## Irisin in Energy Homeostasis and Metabolic Syndrome

Irisin partially bridges the knowledge gap on the interactions of working tissues with other tissues to mediate energy homeostasis. Although irisin is primarily known as a myokine, it is also released from adipose tissue (Moreno-Navarrete et al., 2013; Roca-Rivada et al., 2013), earning its name as an adipokine. It mediates the beneficial metabolic effects of exercise (Grygiel-Górniak and Puszczewicz, 2017). Irisin induces the expression of mitochondrial uncoupling protein 1 (UCP1) and conversion of white to brown adipose tissue, resulting in raised energy expenditure by increased thermogenesis (Zhang et al., 2017). Thus, irisin holds promise to be a therapeutic molecule in mitigation of metabolic syndrome and related disorders.

**FIGURE 2:**
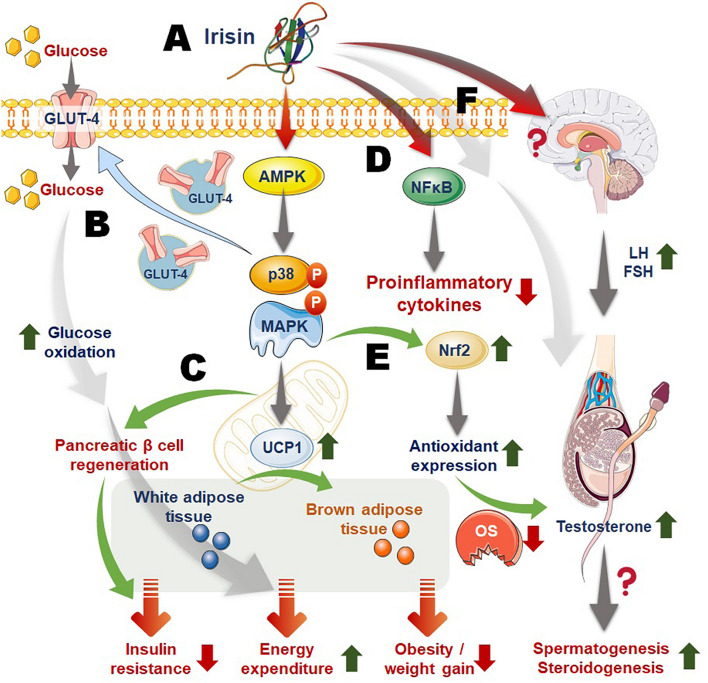
Mechanism of irisin actions linking energy homeostasis, obesity, inflammation, and male reproduction. **(A)** Irisin acts via activation of the AMP activated protein kinase (AMPK), P38, MAPK (mitogen activated protein kinase) pathway; **(B)** irisin-activated pathway upregulates glucose transporter 4 (GLUT4) expression and transportation to membrane, aiding cellular glucose uptake that follows increased glucose metabolism and energy expenditure; **(C)** irisin also induces the expression of mitochondrial uncoupling protein 1 (UCP1) that aids conversion of white adipose tissue to brown adipose tissue, resulting in raised total body energy expenditure, as well as facilitates pancreatic β-cell regeneration that contribute to irisin-mediated reversing of insulin resistance; **(D)** irisin downregulates nuclear factor kappa-B (NF-kB) thereby playing role in suppressing inflammatory responses; **(E)** irisin-induced activation of Nrf2 (nuclear factor erythroid-2 related factor) may increase production of antioxidant enzymes thereby curbing excess reactive oxygen species (ROS) and oxidative stress (OS); **(F)** irisin may act on the HPG (hypothalamic-pituitary-gonadal) axis or directly upon the testicular cells to regulate male reproductive functions. Moreover, irisin actions to improve metabolic balance as well as to reverse obesity, inflammation and OS, may confer ameliorative impact upon obesity/inflammation/OS-mediated male infertility.

Irisin aids skeletal muscle glucose uptake, facilitate glucose and lipid metabolism in liver, serving as insulin sensitizing hormone and reversing conditions of hyperlipidemia and hyperglycemia in metabolic disorders (Chen et al., 2016). Recent studies depicted that irisin stimulates glucose uptake in muscle cells via the calcium/ROS and P38/AMP activated protein kinase (AMPK) mediated pathway (Mu et al., 2001; Zhang et al., 2014; Figure 2). Irisin can ameliorate insulin resistance (IR) by its influence on the functions of the tissues, mainly liver and pancreas, that are involved in the etiology of type 2 diabetes (Moreno-Navarrete et al., 2013; Chen et al., 2016). Moreover, thyroid hormones play significant roles in metabolism, and it is reported that triiodothyronine (T3) can increase the levels of adiponectin and leptin as well as improve insulin sensitivity, while irisin has least impact on adipokines levels, but it plays role in prevention of obesity or body weight regulation owing to its effects upon lipid profile (De Oliveira et al., 2020). Despite, the fact that central irisin administration inhibits the hypothalamic-pituitary-thyroid axis, it appears to be a key regulator of food intake and energy metabolism (Tekin et al., 2018). As irisin impacts on hypothalamic-pituitary-thyroid axis, it indirectly may impact on reproductive functions (Sengupta and Dutta, 2018).

In mature adipocytes, irisin excites white-to-brown fat conversion by elevating UCP1 and PR/SET Domain 16 (PRDM16) via upregulation of p38 mitogen-activated protein kinase (MAPK) and extracellular signal-regulated kinase (ERK) signaling (Boström et al., 2012; Raschke et al., 2013; Huh et al., 2014; Zhang et al., 2014, 2016a). Several studies examined the link between circulating irisin, adiposity, and obesity in humans but with inconsistent results, which may be explained by the fact that irisin may be involved in compensatory mechanism for altered metabolism in obesity and thereby different metabolic status of the specific obese individual determines its levels. Some studies reported a positive correlation between serum irisin levels, body mass index (BMI) and adiposity (Stengel et al., 2013; Crujeiras et al., 2014b), whereas others found inverse association among the circulating irisin levels, BMI and the amount of fat tissue or could not detect any significant change in circulating irisin levels (Huh et al., 2012; Gouni-Berthold et al., 2013). Positive relation of irisin with fat mass, waist circumference, waist-to-hip ratio and leptin levels have also been evidenced in obese subjects (Stengel et al., 2013; Crujeiras et al., 2014b) while a negative association was shown between irisin and adiponectin (Blüher et al., 2014). Furthermore, irisin levels were significantly reduced following weight loss due to bariatric surgery, an effect attributed to a lower fat-free mass and decreased FNDC5 mRNA expression in skeletal muscle (Huh et al., 2012). On the other hand, the reduction in irisin levels was reversed in patients who regained their original weight (Crujeiras et al., 2014a). This suggests that elevated irisin levels could be a compensatory mechanism for the abnormal metabolism and insulin sensitivity characteristic of obese individuals (Huh et al., 2012). Obesity is characterized by systemic inflammation (Bhattacharya et al., 2020), significant imbalance in cytokine secretion that is a strong predictor of developing IR and type-2 diabetes (Fantuzzi, 2005). In addition to cytokines, the activated toll-like receptor 4 (TLR4) is also strongly associated with IR as it increases TNF-α expression, that in turn affects insulin signaling pathway in muscle and adipose tissue (Könner and Brüning, 2011). Interestingly, irisin treatment suppressed expression of pro-inflammatory cytokines, nuclear factor-kappa B (NF-κB), TNF-α, and IL-6 in a concentration dependent manner. Irisin reduced MCP- 1 expression in the cultured adipocytes which subsequently attenuated migration of macrophages in the presence of irisin. Moreover, irisin induced the phenotypic switching of adipose tissue macrophages from M1 (pro-inflammatory) to M2 (anti-inflammatory) state (Dong et al., 2016). Therefore, FNDC5/irisin expression is associated with some anti-inflammatory markers (Moreno-Navarrete et al., 2013).

## Irisin Energy Homeostasis and Male Reproductive Functions

Report by Kim et al. (2018) upended most of the conflicting data on irisin receptor, but it also raises questions and opens up studies in other fields. Are αV/β5 integrin receptors expressed in the male reproductive tract? If yes, what are their specific functions? Would inhibition of irisin (or FNDC5) or its binding to αV/β5 affect male fertility adversely? (Kim et al., 2018). Albeit available data establishing a link between irisin and male reproductive function is scarce, its secretion in the seminal vesicle, penis, and testis (Huh et al., 2012; Aydin, 2014) might infer that it exerts some autocrine and paracrine effects on the male reproductive function. In addition, since energy balance has been established to play key roles in maintaining optimal reproductive function, irisin-driven energy homeostasis may be beneficial to the male reproductive function (Figure 2).

## Irisin and Steroidogenesis

The hypothalamic-pituitary-gonadal (HPG) axis is the main endocrine regulator of the male reproductive functions (Akhigbe et al., 2020). The hypothalamic signal to the pituitary gland is via the gonadotropin-releasing hormone (GnRH) (Dhillo et al., 2005; Corradi et al., 2016). The pituitary gland sends signal to the testis by releasing follicle stimulating hormone (FSH) and luteinizing hormone (LH) from the pituitary gonadotrophs. FSH and LH exert their effects on the testis by binding to FSH-R that is predominantly expressed in the Sertoli cells within the seminiferous tubules and LH-R that is expressed in the interstitial Leydig cells, respectively (Ramaswamy and Weinbauer, 2014). In response to LH signaling, the conversion of cholesterol into testosterone through series of biochemical events (Akhigbe et al., 2020). The gonadotropins establish the adult population of Sertoli, Leydig and stem germ cells and their functions, thus maintaining normal spermatogenesis (Ramaswamy and Weinbauer, 2014). Energy dyshomeostasis has been associated with upregulation of estrogen receptor expression in the male hypothalamus (Chimento et al., 2014). In turn, this triggers a negative feedback mechanism and inhibits the pulsatile release of GnRH, resulting in decline in FSH and LH release and impaired testosterone production. Energy dyshomeostasis also increases the level of aromatase, which raises the conversion of testosterone to estrogen, thus inhibiting testicular function and suppressing circulatory androgen (Hammoud et al., 2006).

Irisin (and FNDC5), possibly via elevation of expression of mitochondrial UCP1, activates thermogenesis and lipolysis with resultant maintenance of energy balance. This might downregulate the expression of estrogen receptor in the hypothalamus, ensuring optimal pulsatile GnRH release and consequent FSH and LH release into the circulatory, resulting in Leydig cell-dependent testosterone production. Re-establishment of energy balance by irisin may also inhibit the conversion of testosterone to estrogen via repression of aromatase activity. Irisin-led elevation of UCP1 and PR/SET Domain 16 (PRDM16) via upregulation of p38 MAPK and ERK signaling (Boström et al., 2012; Huh et al., 2014; Zhang et al., 2014, 2016a) may not only cause white-to-brown fat conversion, but also blunt estrogen-induced cytokine-mediated inflammation.

It is possible that irisin-induced upregulation of p38 MAPK and ERK signaling activates nuclear factor erythroid-2 related factor (Nrf2) (Zhao et al., 2014), resulting in increased expression and activities of antioxidants and protection against ROS attack, oxidative stress and inflammation of the testis (Copple et al., 2017; Askari et al., 2018). Thus, it preserves testicular integrity and function, and promoting testosterone production by mitigating effects of oxidative stress (OS), which has been reported to induce inflammation, and vice versa (Akhigbe et al., 2020), with consequent apoptosis of the testicular tissue and testicular dysfunction.

Irisin may also exert regulatory role on the HPG axis. Kisspeptins, a family of peptides encoded by the KISS1 gene, has been reported to be expressed in the hypothalamus and testis among other tissues (West et al., 1998; Pinilla et al., 2012). The KISS1/GPR54 system plays a central role in the initiation of HPG axis, testosterone production, pubarche, and fertility maintenance (Navarro and Tena-Sempere, 2011). Kisspeptin system thus governs the HPG axis. Impaired expression of the kiss1 gene results in metabolic dysfunction and hypogonadism (Castellano et al., 2006). Reports suggest significant role of kisspeptin neuronal network in connecting energy homeostasis to the reproductive axis (Brown et al., 2008; Hameed et al., 2011). However, the exact mechanism of kisspeptin signaling is unclear. Moreover, kisspeptin signaling has also has a regulatory role in adipose tissue metabolism and it has been found to trigger irisin release (Shamas et al., 2019). Reports also showed that administration of irisin and kisspeptin increased neuropeptide Y (NPY) levels (Ferrante et al., 2016; Orlando et al., 2018) depicting the role of NPY in linking the kisspeptin and irisin pathways. Increase in irisin levels following kisspeptin administration also validate kisspeptin-mediated irisin release via direct irisin neurons stimulation in the hypothalamus or by the active skeletal muscles. It is also being suggested that the interactions between irisin and kisspeptin neurons are involved in regulation of reproductive functions (Tekin et al., 2019). The available reports suggest that irisin, when administered alone trigger reproductive hormones (Jiang et al., 2017), but mediate reverse effects when combined with other factors (such as GnRH and insulin) (Poretsky et al., 2017). Current studies are insufficient to elucidate the effects of irisin on the HPG axis and thus on reproductive functions. Current research is thus not enough to elucidate the impacts of irisin upon the HPG axis with reports claiming irisin to be inhibitory (Poretsky et al., 2017; Tekin et al., 2019), activator (Jiang et al., 2017), or ineffectual (Huh et al., 2014) on reproductive endocrine axis.

## Irisin, Spermatogenesis, and Sperm Quality

Energy dyshomeostasis-led androgen suppression adversely affects spermatogenesis via suppression of testosterone (Bieniek et al., 2016). This results in oligozoospermia and azoospermia (Sermondade et al., 2013). Irisin-mediated upregulation of the expression of Elov13, Cox7a, and Otop1 genes and increased energy expenditure maintain energy homeostasis (Boström et al., 2012). It has been shown that irisin administration in obese male rats could downregulate IR, decrease BMI, enhance the serum levels of FSH and LH, increase testosterone levels thereby resulting in improved spermatogenesis and increased sperm parameters, namely sperm count and motility (Nanees and Reham, 2018). Moreover, *in vitro* study demonstrated the possible role of irisin in spermatogenesis owing to increased irisin expressions in Sertoli cells and undifferentiated spermatogonia transcripts in organotypic primate testicular tissue culture (Wahab et al., 2020).

Several studies have linked obesity with adverse male fertility profile (Jensen et al., 2004; Sermondade et al., 2013). The obesogenic environment stimulates various adipose tissue-derived hormones, among which leptin is widely studied and rise in leptin following energy imbalance leads to increased circulatory estrogen levels, resulting in increased conversion of androgen to estrogen, thereby reducing testosterone levels (Fantuzzi, 2005; Sengupta et al., 2019a). It also reduces sex hormone-binding globulin production (Tsai et al., 2004) thereby restricting the availability of free testosterone. Obesity also mediates increase in pancreatic insulin production and peripheral tissue insulin resistance (Farooqi et al., 2003). Reports claim that obesity-induced altered testosterone production, spermatogenesis and semen quality may be carried out via some common mechanisms that involve OS (Sengupta et al., 2019a). The membranes of the sperm cells are rich in polyunsaturated fatty acid that predisposes them to reactive oxygen species (ROS) attack and oxidative damage including damaged sperm DNA integrity (Selvam et al., 2020). It is credible to suggest that irisin-induced activation of Nrf2 via upregulation of p38 MAPK and ERK signaling (Zhao et al., 2014) may confer protection against ROS attack and oxidative damage to the testis and the sperm cells, thereby enhancing spermatogenesis and sperm quality (Figure 2). Thus, the rise in energy consumption and thermogenesis along with the Nrf2 signaling induced by irisin (Zhang et al., 2014; Askari et al., 2018) would likely cause a decline in energy dyshomeostasis-driven rise in obesity-led oxidative damage. However, studies are needed to validate the most likely assumption that the above mentioned irisin signaling pathway may result in improved insulin sensitivity and sex hormone-binding globulin production, restore testosterone production and functions, spermatogenesis as well as semen quality.

## Conclusion

Irisin is an important novel molecule to be investigated for regulation of metabolic syndrome-induced male infertility. This article describes the major elements of irisin functions and discusses the relevance of irisin in energy homeostasis and male reproduction. Irisin can reverse the adversities of metabolic syndrome-mediated disruptions of male fertility and ameliorates spermatogenesis and steroidogenesis, possibly via its direct and/or indirect beneficial impact of amending insulin resistance, inflammation, OS, imbalanced HPG axis and testicular functions. It thus may provide new approach to treat male reproductive disorders by addressing the root causes of infertility. In-depth investigations are needed to reveal the detailed irisin signaling pathways in regulation of male reproductive functions. While irisin holds high promise in bridging the knowledge gap between energy homeostasis and male fertility various facets await to be explored to show its full potential as a key molecule in reverting metabolic syndrome-induced male reproductive dyshomeostasis.

## Author Contributions

SD, PS, and IK contributed to design the review and conceived the study. SD, PS, IK, RA, and SC drafted, edited, and reviewed the manuscript. SC procured the grant for the publication. All the authors have given their consent for submission.

## Conflict of Interest

The authors declare that the research was conducted in the absence of any commercial or financial relationships that could be construed as a potential conflict of interest.

## Publisher’s Note

All claims expressed in this article are solely those of the authors and do not necessarily represent those of their affiliated organizations, or those of the publisher, the editors and the reviewers. Any product that may be evaluated in this article, or claim that may be made by its manufacturer, is not guaranteed or endorsed by the publisher.
